# Huge scalp Morel-Lavallée lesion with eye involvement in a 3-year-old girl: a case report

**DOI:** 10.1186/s13256-021-02891-6

**Published:** 2021-07-19

**Authors:** Claude Kasereka Masumbuko, Gabriel Kambale Bunduki, Mupenzi Mumbere

**Affiliations:** 1 Department of Surgery, Cliniques Universitaires du Graben, Faculty of Medicine, Université Catholique du Graben, Butembo, Democratic Republic of Congo; 2 Department of Infectious Diseases, Faculty of Medicine, Université Catholique du Graben, Butembo, Democratic Republic of Congo; 3 Department of Paediatrics, Cliniques Universitaires du Graben, Faculty of Medicine, Université Catholique du Graben, Butembo, Democratic Republic of Congo

**Keywords:** Closed degloving injury, Morel-Lavallée lesion, Eye, Scalp, Conjunctival chemosis, Case report

## Abstract

**Background:**

Morel-Lavallée lesions are posttraumatic, closed degloving injuries in which the skin and subcutaneous tissue are separated abruptly from superficial underlying fascia. This condition leads to an effusion containing hemolymph and necrotic fat. Magnetic resonance imaging, when available, is the modality of choice in the evaluation of Morel-Lavallée lesion. Early diagnosis and management is essential as any delay in diagnosis or missed lesion will lead to the effusion becoming infected or leading to extensive skin necrosis. We present a condition of a Morel-Lavallée lesion involving the scalp and complicated by conjunctival chemosis.

**Case presentation:**

We report on a 3-year-old black African girl who presented a fluctuant swelling of entire scalp, extending to upper part of the face on the seventh day after a forehead trauma due to falling on a rock while playing. Skull x-ray revealed soft-tissue swelling, giving an impression of large fluid collection in the deep subcutaneous tissues with no bone fracture. A diagnosis of Morel-Lavallée lesion of the scalp complicated by conjunctival chemosis was made. The patient was managed with percutaneous drainage and compression bandage. The patient improved well and was subsequently discharged without any vision impairment. There was no recurrence of the lesion on follow-up.

**Conclusions:**

The Morel-Lavallée lesion of the scalp complicated with conjunctival chemosis is a rare presentation of this condition. Prompt diagnosis and management are crucial for preventing complications. Image-guided diagnosis and treatment still remain a challenge in the setting of low-resource health facilities.

## Background

Morel-Lavallée lesion (MLL), first described in 1853, is a closed degloving soft tissue injury, a result of abrupt separation of skin and subcutaneous tissue from the underlying fascia [1]. In this condition, there is collection of hemolymphatic liquid in between the two separated layers [2]. Initially, MLL was used in reference to injuries involving the trochanteric region and proximal thigh. However, in recent years, this term has been used for describing lesions with similar pathophysiology in various anatomical locations [3]. MLL diagnosis is mainly based on clinical features and should be taken into account in every traumatic setting. Suspicion should arise in the case of localized swelling, especially with fluctuation. After clinical suspicion, imaging investigations should be performed. Magnetic resonance imaging (MRI) is the modality of choice for investigation of MLL [4]. To prevent infection or extensive skin necrosis as complications, early diagnosis and management of the lesion is critical.

Here we report a rare presentation of MLL involving the scalp complicated by conjunctival chemosis in a 3-year-old girl. To our knowledge, this is the first case of MLL with eye involvement reported in the literature.

## Case presentation

**Fig. 1:**
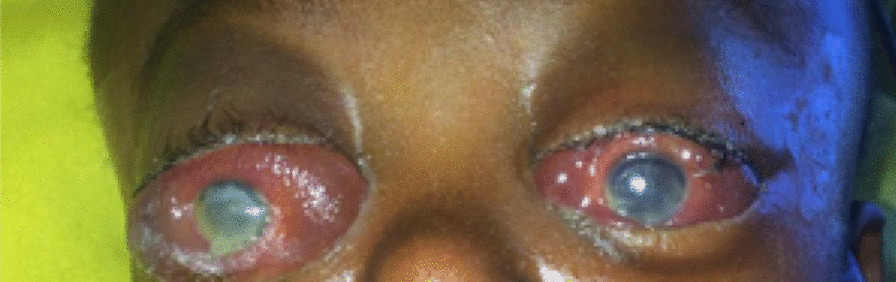
Fluctuant swelling in eye and scalp

**Fig. 2:**
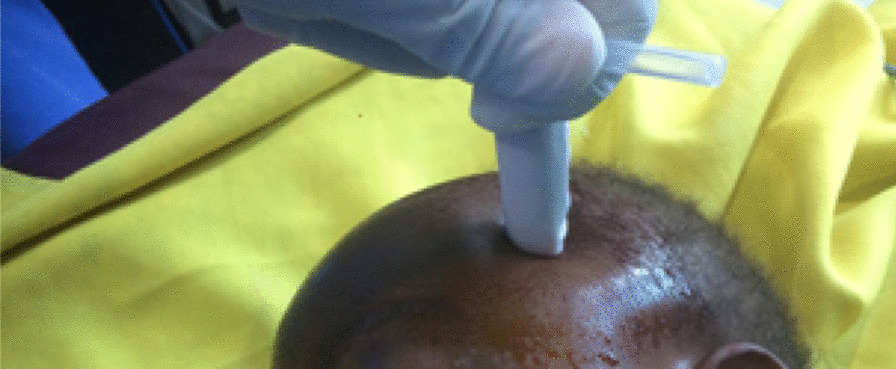
Drainage of the hemolymphatic liquid

**Fig. 3:**
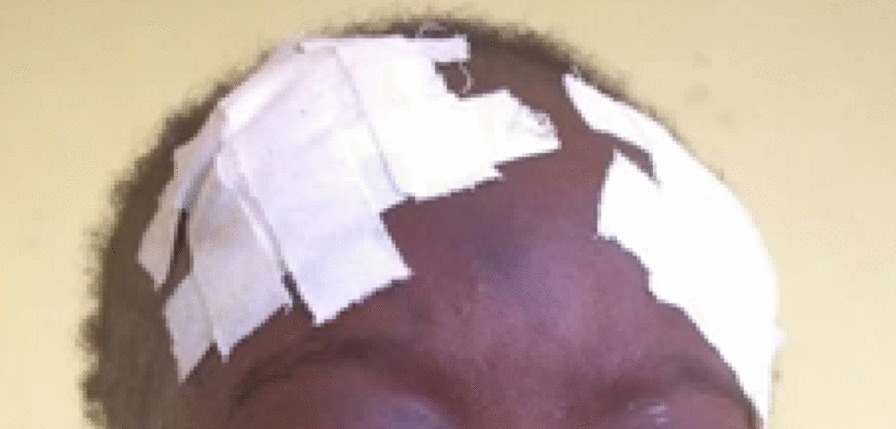
Patient with the compression bandage

A 3-year-old black African female was brought to our emergency room with trauma to the forehead and history of fall on a rock while playing. There was a delay of 7 days between the trauma and presentation to the health facility. No history of loss of consciousness was mentioned. Admitted to a nearby medical facility, the daily increase of head circumference with the protrusion of eyeballs and eye watering motivated the facility to refer the patient. On clinical examination, she had palpebral conjunctiva protrusion, fluctuant swelling of entire scalp extending to upper part of the face (Fig. 1). There was no skin necrosis. Skull x-ray demonstrated soft tissue swelling, giving an impression of a large fluid collection in the deep subcutaneous tissue with no bone fracture observed. Ultrasound (US) of the fluctuant swelling in the scalp was difficult to conduct. No MRI machine was available. Ophthalmologic examination revealed conjunctival chemosis and blurred vision. Laboratory tests revealed the manifestations of infected lesions [hemoglobin level of 7 mg/dl and white blood cells (WBC) count of 29,800/mm^3^]. The diagnosis of Morel-Lavallée lesion with conjunctival chemosis was established. The patient received two pints of packed red blood cell (PRBC) transfusion and ceftriaxone 350 mg intravenously twice a day. She was also managed with percutaneous drainage and compression bandage (Figs. 2, 3). The lesion subsided at the time of discharge with full visual capacity recovery. There was no recurrence of the lesion on follow-up.

## Discussion and conclusions

The diagnosis of MLL is challenging owing to its inconsistent clinical presentation and the initial involvement of skin bruising due to underlying soft-tissue injury. Clinical presentation varies according to the amount of hemolymphatic liquid collected at the site of injury and the time elapsed since the injury [2, 5]. The symptoms include soft-tissue swelling giving the impression of a soft fluctuant area, cutaneous sensation decreased, skin hypermobility, contour deformity, palpable bulge, etc. [6]. In our case, these symptoms were extended to both eyes, giving conjunctival chemosis. Several published studies have reported cases of MLL involving lumbar area, hip, and inferior members [7–9], though none has described MLL with eye involvement. To our knowledge, this is the first reported case in the literature.

Since diagnosis of MLL is challenging owing to inconsistent clinical presentation, imaging investigations are very useful. Radiological findings are characterized by the presence of nonspecific and noncalcified soft-tissue mass. Meanwhile, US scan may reveal hyperechoic (blood-predominant) or anechoic (lymph-predominant) images depending on the age and predominant content of the lesion. A heterogeneous image with irregular margins and lobular shape may be seen in lesions less than 1 month old. For lesions older than 1 month, a homogeneous image with smooth margins and flat or fusiform shape may be seen. MRI scan shows soft-tissue contrast enhancement [4, 10]. Hence, it is the modality of choice in the evaluation of MLL. Due to financial constraints and lack of equipment such as MRI in our setting, the diagnosis was done on the basis of clinical and skull x-ray assessment.

In this case, the conjunctival chemosis and blurred vision may be explained by the conjunctival and probably corneal infiltration of swelling and the inflammatory reaction. To the best of our knowledge, this is the first case of MLL of scalp with eye involvement reported in the literature.

This case was managed with percutaneous drainage and compression bandage. An antibiotic therapy was added to this approach. Currently, there is no accepted management approach for MLL. A variety of conservative and surgical options exist. This may be a stage-based or an individualized management approach. Objectives of management include drainage of the hemolymphatic liquid for resolving the contour deformity and allowing the apposition of lesion walls, debridement for removing necrotic tissues or mature capsule in chronic lesions, and meticulous dead space management to prevent recurrence by facilitating long-term adhesion of lesion walls either by fibrosis or using surgical apposition techniques [11, 12]. In addition to that, a definitive management of associated injuries should be done.

Recently, a new management algorithm was proposed and appeared to be a rational approach [11]. It recommends the distinction of lesions into acute and chronic. An absolute surgical exploration is indicated in the case of acute MLL with open fracture associated with lesion, skin necrosis, and infection. This approach may also be indicated in the case of failure of nonsurgical approach, in chronic symptomatic lesions, and when the lesion is in association with a closed fracture requiring open fixation [11]. Complications associated with MLL occur often as a result of delayed or incorrect diagnosis. Progressive expansion of untreated lesions can cause pressure necrosis of overlying skin. This can result in large areas of skin breakdown and leave underlying fractures exposed [12].

In conclusion, MLL is a rare condition found after a trauma and can lead to infection, skin necrosis, or chronic encapsulated hemolymphatic liquid effusion. This is a rare presentation of MLL with conjunctival chemosis. Diagnosis of MLL can be made on the basis of clinical and radiological examination. Its diagnosis and treatment are often delayed because they involve internal degloving without surface penetration. Image-guided diagnosis and management is a challenge for health facilities in poor settings.

## Data Availability

The datasets used and/or analyzed during the current study are available from the corresponding author on reasonable request.

## Abbreviations

MLL: Morel-Lavallée lesion
MRI: Magnetic resonance imaging
WBC: White blood cells
PRBC: Packed red blood cells
US: Ultrasound scan
